# Assessing the validity of respondents’ reports of their partners’ ages in a rural South African population-based cohort

**DOI:** 10.1136/bmjopen-2014-005638

**Published:** 2015-03-06

**Authors:** Guy Harling, Frank Tanser, Tinofa Mutevedzi, Till Bärnighausen

**Affiliations:** 1 Department of Global Health and Population, Harvard School of Public Health, Boston, Massachusetts, USA; 2 Wellcome Trust Africa Centre for Health and Population Studies, Mtubatuba, KwaZulu-Natal, South Africa

**Keywords:** EPIDEMIOLOGY, PUBLIC HEALTH, STATISTICS & RESEARCH METHODS

## Abstract

**Objectives:**

This study evaluated the validity of using respondents’ reports of age disparity in their sexual relationships (perceived disparity), compared to age disparity based on each partner's report of their own date of birth (actual disparity).

**Setting:**

The study was conducted using data from a longitudinal population-based cohort in rural KwaZulu-Natal, South Africa, between 2005 and 2012.

**Participants:**

The study used 13 831 reports of partner age disparity within 7337 unique conjugal relationships. 10 012 (72.4%) reports were made by women.

**Primary and secondary outcome measures:**

The primary outcome was the Lin concordance correlation of perceived and actual age disparities. Secondary outcomes included the sensitivity/specificity of perceived disparities to assess whether the man in the relationship was more than five or more than 10 years older than the woman.

**Results:**

Mean relationship age disparity was 6 years. On average, respondents slightly underestimated their partners’ ages (male respondents: 0.50 years; female respondents: 0.85 years). Almost three-quarters (72.3%) of age disparity estimates fell within 2 years of the true values, although a small minority of reports were far from correct. The Lin concordance correlation of perceived and actual age disparities (men: ρ=0.61; women: ρ=0.78), and assessments of whether the man in the relationship was more than five, or more than 10 years older than the woman (sensitivity >60%; specificity >75%), were relatively high. Accuracy was higher for spouses and people living in the same household, but was not affected by relationship duration.

**Conclusions:**

Rural South Africans reported their sexual partners’ ages imperfectly, but with less error than in some other African settings. Further research is required to determine how generalisable these findings are. Self-reported data on age disparity in sexual relationships can be used with caution for research, intervention design, and targeting in this and similar settings.

**Strengths and limitations of this study**

- This study provides the first analysis of the validity of individuals’ perception of their partner's age in South Africa, and only the second such analysis in sub-Saharan Africa.
- Self-reported and partner-reported age data are both drawn from a very large longitudinal, population-based cohort.
- The strong vital statistics programme in South Africa, and the provision of dates of birth in identification books provided to all South Africa citizens means that the measurement of age (self-report) in the reference data set is likely to be highly accurate.
- The study sample is drawn from a single population living in a poor community in rural KwaZulu-Natal, South Africa; results may therefore not generalise to settings, such as wealthy or urban communities.
- The relationships included in the analysis were only those involving cohabiting couples; results may therefore not be generalise to other relationship types, such as casual relationships.

## Background

Age disparities in sexual relationships are believed to place younger women at increased risk for various ills, including intimate partner violence, unsafe sexual behaviours and acquisition of sexually transmitted infections (STI).^1–3^ These risks can arise from unequal power dynamics within age-disparate relationships or from an increased likelihood that the older male partner carries risk factors such as psychological traits. In the case of HIV and other STIs, older men are also more likely to be infected, which is of course a pre-requisite for transmitting an infection to their partners.

Research on age disparities and health outcomes has, to date, focused largely on acquisition of HIV or other STIs. Cross-sectional studies have generally found positive associations between age disparity and STIs,^4–6^ although a recent longitudinal study found no association.^7^ Failure to use condoms, a risk factor for STIs and pregnancy, also appears to be higher in age-disparate relationships.^8^ ^9^ Associations between age disparity and intimate-partner violence have been mixed across studies.^10–12^ Concerns regarding health outcomes have led to several campaigns to reduce age-disparate relationships between young women and older men.^13^ ^14^

Research and interventions relating to relationship age disparities typically rely on an individual's perception of their partner's age: in the case of research, in order to measure the disparity; in the case of interventions, to raise individuals’ awareness of relationship age disparity and to initiate actions to reduce it. The reliance on perception in establishing age disparity in research and interventions may be problematic. Demographers have long recognised that reporting of one's own age suffers from non-random measurement error. Such errors may be unintentional, due to uncertainty arising from poor date of birth knowledge^15^ ^16^ or cognitive biases towards reporting landmark ages (eg, those ending in 0 or 5) leading to ‘heaping’ of disparity data.^17^ ^18^ In addition, intentional reporting biases may arise from efforts to meet age-eligibility cut-offs,^19^ or from social desirability concerns, either relating to one's absolute age, or relationship age disparity.^20^ ^21^ In consequence, self-reported and partner-reported ages may both suffer from error.

Current evidence on the validity of reports on sexual partners’ ages is very limited. One study from the USA suggested that partners report one-another's age accurately.^22^ However, a study in Malawi found very poor sensitivity among women identifying whether their male partners were more/less than 5 or 10 years older than themselves.^23^ One reason suggested for the Malawian finding was the lack of systematic vital registration,^24^ which leaves many without a formal record of their date of birth.

We analyse the validity of individuals’ reports of their conjugal partners’ ages in a rural South African setting, in KwaZulu-Natal province. KwaZulu-Natal recently began a social marketing campaign against age-disparate partners, aiming to prevent HIV infection and teenage pregnancy.^13^ Given South Africa's interest in age disparities, and the high burdens of HIV^25^ and unplanned pregnancy,^26^ evidence of accuracy of reported age disparity in the country is timely. Additionally, South Africa provides a setting in which to test the contributions of intentional and unintentional misreporting since, in contrast to other African settings, date of birth information is broadly available in South Africa. The date of birth is the first six digits of each person's national identification number, contained in the national identification book.

We thus test, for the first time in South Africa, the accuracy of respondents’ assessments of their main sexual partner's age. Our results will inform the extent to which researchers can rely on partner age reports for analysis, and the extent to which those planning behaviour change interventions can rely on individuals to accurately assess their exposure to age-disparate relationships.

## Methods

We used data from the Africa Centre Demographic Information System (ACDIS), a longitudinal, population-based open cohort maintained through a demographic surveillance site, the Wellcome Trust Africa Centre for Health and Population Studies, which is located in a predominantly rural area of the uMkhanyakude district, KwaZulu-Natal.^27^ Since 2000, ACDIS has been collecting household demographic data from a key informant in each household, initially two and now three times per year. When an individual is first registered, their date of birth is requested to the greatest accuracy known (eg, day, month, quarter, year) and the source of this information (national identification number, seen by interviewer; national identification, unseen by interviewer; memory, self; memory, other person) recorded. A record is also made at each demographic surveillance visit of the start or end date of any new conjugal relationships formed or ended by women, again to the greatest accuracy known, as well as whether the relationship is marital. (Conjugal relationships are defined as “married or regular sexual partners who are members of the same household, regardless of their place of residence”.^28^) Conjugal relationship records allow the linkage of each partner to the ACDIS database, which includes a range of data on demographic, health, economic and behavioural variables.

Since 2005, ACDIS has invited all those aged over 15 to respond to an annual general health survey, which includes sexual history questions. For each of their three most recent sexual relationships, respondents report: how much older or younger the partner is (measured in single years); the relationship type (spouse, regular partner, casual partner or previous spouse/regular partner); and the date of last sex, if the relationship has ended. These questions are asked independently of the conjugal relationship records. Within the entire ACDIS, approximately 15% of partners are spousal, 65% regular, 5% casual and 15% previous partners. At each general health interview individuals are also asked to verify their date of birth; if it differs from the recorded value, the new date and its source replace any previous value reported by another household member. In our data set, 6.6% of respondents updated their date of birth between 2005 and 2013; this figure was significantly higher in younger age groups but did not differ by sex (see online supplementary table S1). Ownership of a national identification book is very high in this community. The recorded source for date of birth for almost all (>99%) of our analytic sample respondents and their partners was thus the national identification number—either seen or unseen.

The population for this analysis was all non-casual relationships reported in the general health surveillance between 1 January 2005 and 31 December 2012 by individuals aged 15 years or older. The same relationship could be reported in multiple years and multiple relationships could be reported each year. We matched each relationship report to a single conjugal relationship based either on the date of interview, or the date of last sex if the relationship had ended. When more than one conjugal relationship was ongoing at the relevant date, we used an algorithm to identify whether one match was considerably more likely than the other(s) (see online supplementary appendix 1). Our analytic sample included all general health relationships to which a single conjugal relationship could be uniquely matched and for which the respondent reported an age disparity (including those reporting no difference in age). We excluded anyone whose birth year was unknown (we ran subanalyses including only individuals whose date of birth was known (1) within a month; and (2) to the day).

Our measure of interest was age disparity between the respondent and their partner. This was measured in two ways. Our reference data set was the difference between the respondent's self-reported age and their partner's self-reported age (hereafter the ‘actual’ disparity). The comparison measure was the respondent's report of age disparity in their relationship (hereafter the ‘perceived’ disparity). Using self-reported age as the reference measure was appropriate given the ubiquity of national identification numbers. Since 1992, identification numbers have been issued as part of the national birth registration process. Birth certificates are issued directly at regional hospitals or based on hospital or midwife-issued birth records for other births. The identification number is then transferred from the birth certificate to the identification book, which is issued from age 16 onwards. Prior to 1992, birth certificates did not contain the identification number and were commonly only obtained several years after birth—often in order to attend school. Late applications for birth certificates required the production either of the original hospital record for the birth, or written testimony by a traditional leader (in a rural area) attesting that the child was known to be the woman's child and stating the date of birth, discerned using event calendars.

Nationally, the proportion of births registered in the year of birth rose from 46% in 1992 to 79% in 2012; and by the end of this period registration completeness within 5 years was 98%.^29^ In uMkhanyakude district, completeness of birth registration in the same year was 73% in 2012, with more than half of all late registrations occurring in the subsequent year. These data suggest that while errors in dates of birth are likely to be present in some national identification numbers, particularly those for older individuals, the majority of national identification numbers are an accurate reflection of a person's year of birth.

### Statistical analyses

All analyses were stratified by respondent's sex. We first measured the distributions of actual and perceived age disparity, and of the difference between them. We computed two concordance measures appropriate for validating a measurement variable (perceived age disparity) against a reference (actual age disparity). The Bland-Altman procedure provides a visual display of concordance and 95% limits of agreement between two measures, that is, a two standard-deviation range between which 95% of observed differences are expected to lie.^30^ The Lin concordance correlation coefficient measures agreement by correcting the Pearson correlation coefficient for bias that arises from systematic difference in the values of one measure compared to the other (eg, if one approach consistently provides higher values).^31^ We also estimated the sensitivity and specificity of the perceived age disparity in identifying relationships in which the man was 5 or 10 years older than the woman.

We conducted bivariate regression of the absolute difference in age disparity measures on characteristics of the respondent–respondent age (15–24, 25–34, 35–49, >49 years old); number of partners in past 12 months (0, 1, >1)—and of the relationship—type of relationship (current partner, current spouse, former partner/spouse); time involved in relationship; and whether the partner was currently a member of the household. We imputed missing covariate values 20 times via chained equations, using all analytic covariates plus age at first marriage, age at first sex, time since last sex, lifetime number of partners and use of condom at last sex. These imputed data were used only in the bivariate regression models.

Our data set contained many relationships with repeated evaluations of age disparity. We hypothesised that respondents’ accuracy in evaluating partner age would improve as relationships continue. Therefore, for all relationships in which partner age disparity was reported more than once, we reran our analyses of concordance and sensitivity/specificity for the first and the last age report, and conducted bivariate fixed effects regression of time since first interview on accuracy for all available reports. Additionally, given concerns that respondents may heap age reports,^17^ ^18^ we measured the degree of age heaping in self-reported age and age disparities measured based on each partner's self-reported age as well as on the age disparity report of each individual.^32^ ^33^

Informed consent is required separately for the demographic and sexual health questionnaires. This analysis was exempted from additional review by the Harvard School of Public Health Institutional Review Board due to its exclusive use of anonymised secondary data.

## Results

Between 2005 and 2012, 17 440 non-casual relationships reports were made through the general health survey by respondents who were in concurrent conjugal relationships. We were able to uniquely match a conjugal relationship at the relevant date in 16 638 (95.4%) cases. Of these relationships, age disparities were reported in 13 894 (83.5%) cases. In only 63 (0.5%) cases was either partner's date of birth less precise than 1 year. Our primary data set was thus formed of 13 831 reports from 7337 unique relationships. The data set comprised 3819 (27.6%) male and 10 012 (72.4%) female responses and the median relationship period was long—10 years for men, 8 for women (table 1). Missingness for covariates was low, ranging from 0.1% for ‘number of partners in past year’ to 9.9% for ‘time sexually involved’.

**Table 1:** Characteristics of sexual partnerships

	Sex of respondent			
	Male		Female	
Sample size	3819	(27.6%)	10 012	(72.4%)
Age of respondent (years)				
15–24	221	(5.8%)	810	(8.1%)
25–34	989	(25.9%)	2988	(29.8%)
35–49	1889	(49.5%)	5585	(55.8%)
>49	720	(18.9%)	629	(6.3%)
Age of partner, reported (years)				
15–24	614	(16.1%)	253	(2.5%)
25–34	1245	(32.6%)	1828	(18.3%)
35–49	1575	(41.2%)	5303	(53.0%)
>49	385	(10.1%)	2628	(26.2%)
Age of partner, actual (years)				
15–24	581	(15.2%)	169	(1.7%)
25–34	1215	(31.8%)	1685	(16.8%)
35–49	1590	(41.6%)	5291	(52.8%)
>49	433	(11.3%)	2867	(28.6%)
Age disparity, actual (mean, SD)	−4.5	(5)	5.9	(6)
Age disparity, reported (mean, SD)	−4.0	(5.7)	6.7	(6.8)
Difference between reported and actual age disparity (mean, SD)	−0.50	(4.7)	−0.85	(4.2)
***Respondent characteristics***				
Number of partners in past 12 month (mean, SD)	1	(0.42)	1	(0.37)
Missing	13	(0.3%)	7	(0.1%)
***Partnership characteristics***				
Relationship type				
Current partner	2222	(58.2%)	4352	(43.5%)
Current spouse	1279	(33.5%)	4406	(44.0%)
Former partner/spouse	318	(8.3%)	1254	(12.5%)
Partner member of household	2938	(76.9%)	7912	(79.0%)
Missing	22	(0.6%)	29	(0.3%)
Months sexually involved (median, IQR)	120	(60, 216)	96	(107, 168)
Missing	357	(9.3%)	1006	(10.0%)
Days since last sexual intercourse (median, IQR)	7	(2, 21)	10	(3, 35)
Missing	416	(10.9%)	781	(7.8%)

All figures are numbers and per cent unless otherwise noted.

On average men were older than women, with larger mean disparities for female-respondent (6.7 years) than for male-respondent (4 years) relationships. Respondents slightly underestimated their partner's age, women by a mean of 0.85 years and men by 0.50 years; the median difference for both sexes was −1 year and this was statistically significant for women (Wilcoxon sign-rank Z=−28.8, p<0.001) and also for men (Z=−15.6, p<0.001).

**Figure 1:**
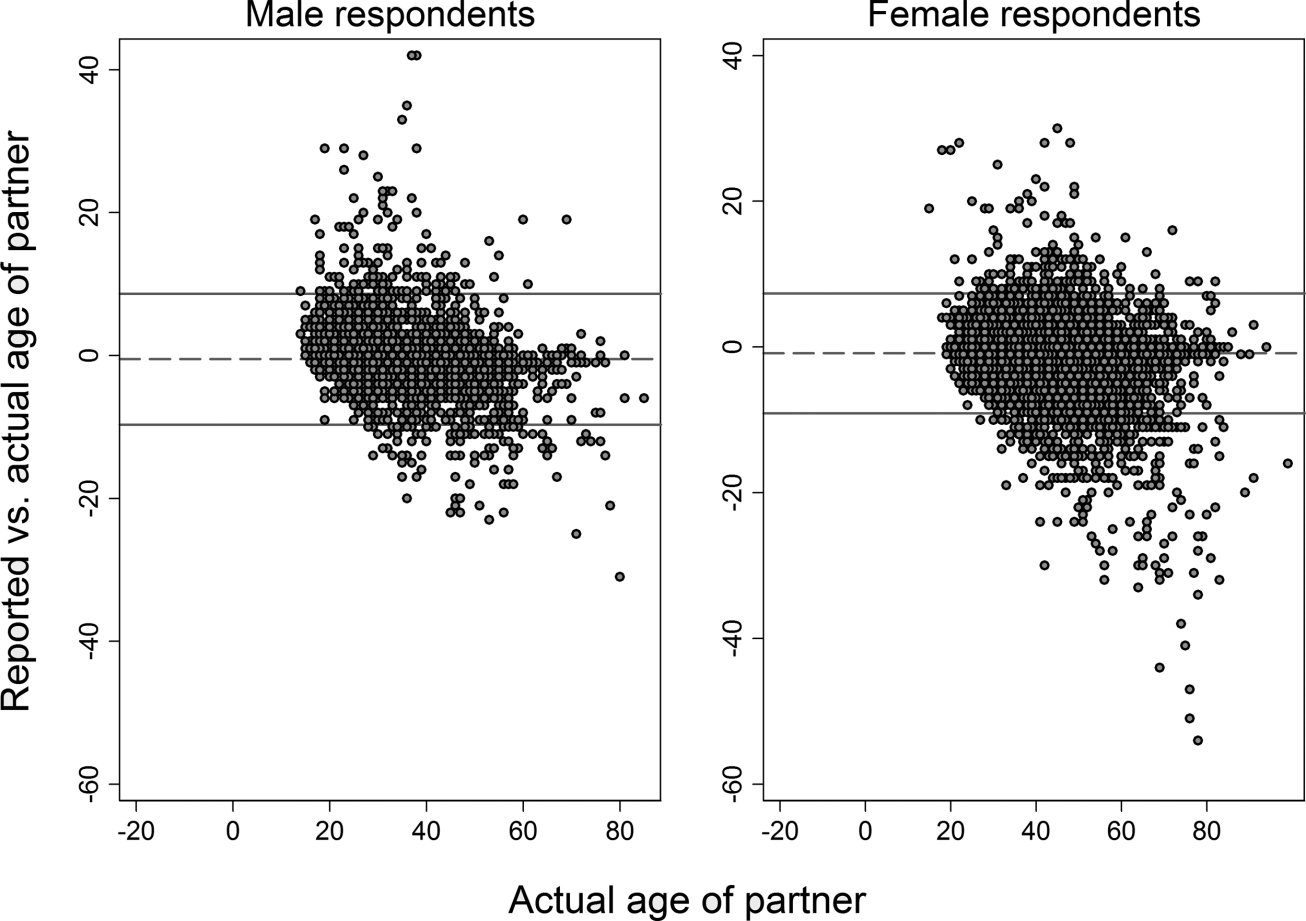
Distribution of difference in years between reported and actual age disparity in conjugal relationships, stratified by respondent's sex (n=13 831).

**Figure 2:**
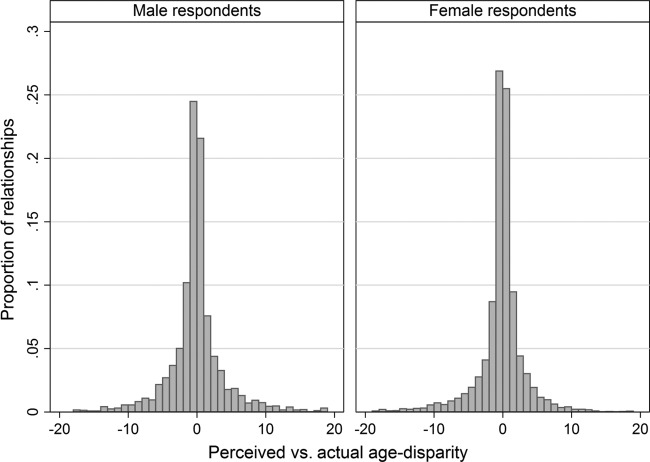
Bland-Altman plots of agreement for reported and actual partner ages, stratified by sex of respondent. Mean difference indicated by dashed horizontal line; 95% limits of agreement indicated by solid horizontal lines.

Over half of respondents (59.1%) reported their partner's age to within 1 year of the truth, and 72.2% were within 2 years (figure 1). However, a sizeable minority of respondents reported partner ages far from the truth: Bland-Altman plots found similar 95% limits of agreement for male and for female respondents: −9.7 to 8.7 for men; −9.1 to 7.4 for women (figure 2). A small minority of respondents (3%) reported partner ages that differed from the reference age by more than 10 years. Jointly, these figures highlight the strong impact on overall variance of a small number of reports that were highly inaccurate.

The narrower range of limits of agreement for women reflects lower variance in accuracy for women than for men; this finding is supported by the higher Lin concordance correlation coefficient for female (ρ=0.78, 95% CI 0.77 to 0.79) compared to male (ρ=0.62, 95% CI 0.60 to 0.64) respondents. The accuracy of responses also varied within sex by age of respondent: men's as well as women's mean error decreased with age, although variance in accuracy rose steeply for men but not for women (see online supplementary table S2).

In over half of all relationships reported (52.9%), the male partner was five or more years older (table 2). Sensitivity and specificity for respondents’ capacity to identify whether or not they were in such a relationship was over 75%, for men as well as for women. For relationships in which the man was 10 or more years older, sensitivity was lower but specificity was higher, reflecting the lower prevalence of such relationships (21.1%). The positive and negative predictive values of the data set varied between 60% and 94%, and the area under the receiver-operating characteristic curve was 78% for men and 84% for women at both age disparity cut-points.

**Table 2:** Sensitivity and specificity for reports of age-disparate relationships

	Man ≥5 years older				Man ≥10 years older			
	Male respondent		Female respondent		Male respondent		Female respondent	
Prevalence (%)	40.2		57.8		13.9		23.8	
Sensitivity (%)	78.1	(75.9–80.1)	79.6	(78.5–80.6)	61.6	(57.3–65.7)	72.6	(70.7–74.4)
Specificity (%)	79.2	(77.4–80.8)	88.5	(87.5–89.4)	93.6	(92.7–94.4)	94.8	(94.2–95.3)
Positive predictive value (%)	71.6	(69.4–73.7)	90.4	(89.6–91.2)	60.8	(56.5–64.9)	81.3	(79.5–82.9)
Negative predictive value (%)	84.3	(82.7–85.8)	76.0	(74.8–77.2)	93.8	(92.9–94.6)	91.7	(91.1–92.3)
Area under ROC curve (%)	78.6		84.0		77.6		83.7	

Prevalence: proportion of all relationships that are age disparate at the relevant cut-off. Sensitivity: proportion of truly age-disparate relationships reported as age disparate. Specificity: proportion of truly non-age-disparate relationships reported as non-age-disparate. Positive predictive value: proportion of relationships reported age disparate that truly are age disparate. Negative predictive value: proportion of relationships reported non-age-disparate that truly are non-age-disparate.

ROC, receiver-operator characteristic.

The absolute size of the difference between perceived and actual age disparity declined with age among women, but rose for men, so that reports by those aged over 49 years were over 1 year less accurate than reports by those aged under 25 years (table 3). Similarly, having more sexual partners in the past year was associated with significantly decreased accuracy in perceived age disparity for men, but not for women. Among relationship-specific variables, within-household partners and current spouses were more accurately reported by both sexes (see online supplementary figure S1). Longer involvement increased accuracy slightly: 10 years of additional relationship-time was associated with a 0.17 year improvement for men and a 0.35 year improvement for women.

**Table 3:** Sex-stratified bivariate regressions of association between respondent and relationship characteristics, and absolute difference in years between actual and reported relationship age disparity

	Male respondent		Female respondent	
N	3819		10 012	
*Respondent characteristics*				
Age of respondent* (years)				
15–24	Reference		Reference	
25–34	0.00	−0.55 to 0.56	−0.55	−0.83 to −0.27
35–49	0.45	−0.08 to 0.98	−0.59	−0.86 to −0.33
>49	1.03	0.46 to 1.61	−0.36	−0.74 to 0.02
	Z=1.60, p=0.109		Z=−1.94, p=0.052	
Number of partners in past 12 months	0.30	0.01 to 0.59	−0.13	−0.32 to 0.06
*Relationship characteristics*				
Time sexually involved (years)	−0.02	−0.03 to 0.00	−0.03	−0.04 to −0.03
Partner member of household	−0.66	−0.95 to −0.37	−0.89	−1.06 to −0.71
Relationship type†				
Current partner	Reference		Reference	
Current spouse	−0.48	−0.74 to −0.21	−0.56	−0.71 to −0.41
Former partner/spouse	0.25	−0.20 to 0.69	0.26	0.03 to 0.48
	F_(2)_=8.12, p<0.001		F_(2)_=38.45, p<0.001	

All figures are point estimates and 95% CIs unless otherwise noted.

*Z tests are non-parametric tests for trend across ordered groups, an extension of the Wilcoxon rank-sum test.

†F_(k-1)_ tests are Wald-type tests for difference among all regression coefficients for the independent variable.

Comparisons of agreement measures for the first and last reports for the 3501 multiply reported conjugal relationships found no significant change with increasing time under observation for concordance or sensitivity/specificity (see online supplementary table S3). In fixed effects regression, years since first interview did not have a significant effect on reporting accuracy either for men (0.07 years per additional year of observation, 95% CI 0.00 to 0.15) or for women (0.02, 95% CI −0.01 to 0.05).

Age reports at baseline and age disparities in relationships based on each partner's report of their own age, contained very little age heaping (see online supplementary figures S2a and S2b). Perceived age disparities contained considerable heaping at many 5-year and 10-year intervals (see online supplementary figure S2c). Rerunning our analyses restricted to those whose date of birth was specified to within 1 month (n=12 401; 89.6%) or to the day (n=11 855; 85.7%) did not change our findings (see online supplementary table S4).

## Discussion

We evaluated individuals’ capacity to accurately identify their partner's ages using 8 years of population surveillance data in rural KwaZulu-Natal, South Africa. In this setting, where literacy levels are low by South African standards but high relative to the continent as a whole,^34^ ^35^ individuals’ assessments of the age disparity between themselves and their sexual partners, while imperfect, were more accurate than those seen in rural Malawi,^23^ and achieved moderate levels of discriminatory power.

**Figure 3:**
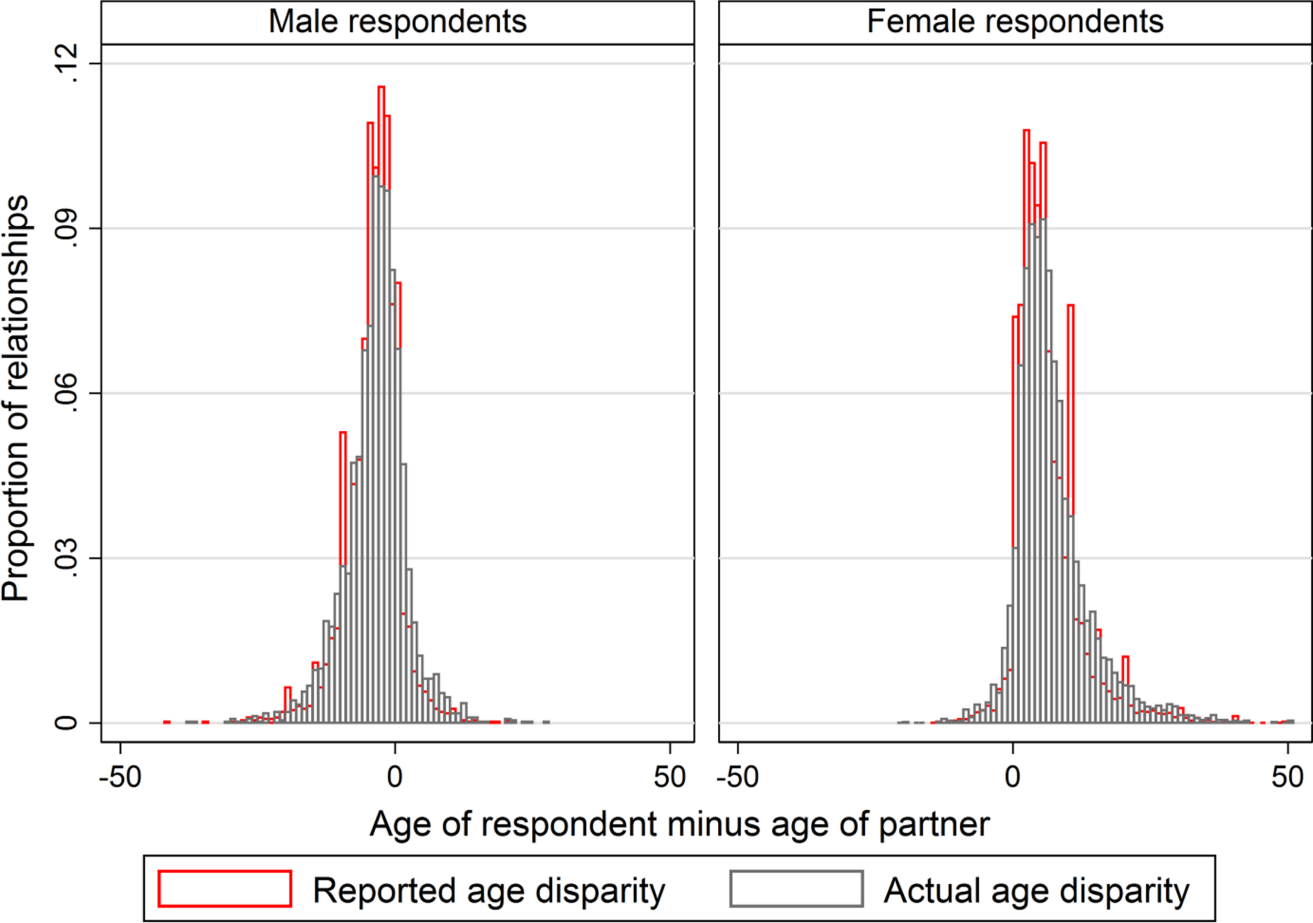
Distribution of reported and actual age disparities (in years), by gender. The proportion of relationships with actual (light grey bars) and reported (dark red bars) age disparities at each age difference are overlaid, so that areas where the red bars are clearly visible (close to zero, and at heaping values such as men being 5 years older) represent age disparities that are reported more often than they actually occur.

In our study, individuals underestimated their partner's age by less than 1 year on average. This average value suggests that there was a slight tendency to over-report age disparities near zero, and to under-report extreme differences (see figure 3). However, the low average does not imply that there are no issues with partner-reported age. First, as reported above, partner-reported ages show overly high levels of reporting of rounded age disparities (ie, ±5, 10, 15 years), in contrast to the smooth distribution of self-reported ages (see online supplementary figure S2). This discrepancy may well reflect the different data collection methods for own and partner age reports. Own age reports are often captured via dates of birth from national identity documents, as described above, and are thus supported by a relatively strong vital registration system. In contrast, reporting a partner's age is potentially complicated by lack of precise knowledge of a partner's age and by the higher cognitive burden of recalling someone else's age. Both of these factors may lead to the use of heuristics for estimating age disparity, and thus age heaping.

Second, in addition to many small inaccuracies in reports of partner ages—three-quarters of individuals reported partners’ ages that fell within 2 years of the age in the reference data set—a small proportion of partner age reports was highly incorrect. Even though rare overall, these highly incorrect reports may be particularly worrying if the likelihood of this type of misreporting differs systematically by other factors that may be of interest in an analysis. In interpreting these misreports it is important to note that our analysis compared data from two independent reports of partners—conjugal relationships and sexual behaviour partnerships—which we matched to one another for our analyses. Some of the extreme inaccuracies we report may therefore reflect a failure to correctly match sexual behaviour reports to conjugal relationships, and thus our results may overestimate the inaccuracy in knowledge of partner age in this population. We have attempted to minimise the risk of mismatching partners by dropping observations where we were unable to clearly identify unique partners of each respondent with similar relationship start dates and durations in both data sets (see online supplementary appendix 1); however, we cannot rule out this source of potential error.

### Implications for choice of dichotomous cut-points for defining relationships by age disparity

Inaccuracy in age disparity reporting has important implications in the context of the common approach of using dichotomised age disparity measures (eg, 5-year or 10-year cut-points for defining ‘age-disparate’, ‘intergenerational’ or ‘sugar daddy’ relationships^1^ ^3^). Small inaccuracies biased towards ‘no disparity’ will tend to lead to an underestimate of the prevalence of age-disparate relationships. This effect will be stronger, the higher the density of relationships with true disparities close to the cut-point. The level of error introduced into dichotomous age disparity measures by small underestimates will be exacerbated by heaping of reports at the very values that are commonly used as cut-points for analysis. The exacerbation arises because heaping typically involves pulling from values close to the heaped value, and thus the cut-point value will contain age-disparate as well as non-disparate relationships, leading to more misclassification than if any other cut-point age were used. For example, if ‘age-disparate’ is defined as ‘5 years or older’, and heaping is common at this value, many relationships where the partner is truly 4 years older will be erroneously classified as age disparate.

In reality, the age heaping seen in this study reflects respondent-driven coarsening of data reporting, over and above the interviewer-driven coarsening generated by asking for age disparity in years, rather than for the partner's date of birth. Both forms of coarsening—from daily to annual, and from annual to quinquennial—are only problematic when the degree of coarseness is large relative to the average disparity or category sizes being measured. Thus, in this setting where average relationship age disparities are several years, respondent age heaping is likely to have less impact on accuracy than in a setting where the great majority of relationships display disparities of 1 year or less.

A key implication of this discussion of empirical age disparity heaping is that a continuous measure of age disparity is likely to suffer from less error in perception and in reporting than a dichotomous one. This data-driven conclusion buttresses the theoretical continuum of effect: for many outcomes, such as HIV acquisition, pregnancy or intimate partner violence, there is no conceptual basis for age disparities of 4 years having no impact at all on the outcomes but for age disparities of 6 years substantially determining outcomes. Rather, the mechanisms driving risk for violence, STIs or pregnancy are likely to rise smoothly with increasing age disparity.

### Variation in results by subpopulation

In our study, accuracy of age disparity reports was higher for current spouses and current members of the respondent's household. However, increased time in a relationship did not increase accuracy, suggesting that perceptions of partner's age are resistant to change once set. Accuracy was higher for female respondents than for men: although the mean difference between ‘actual’ and ‘perceived’ age disparities was larger for women, the variance and IQR were smaller, and the concordance correlation coefficient larger, than for male respondents. Additionally, women's accuracy rose modestly with age, while for men it changed little after 25 years of age. These differing age trends by sex may reflect the interaction of two processes: increasing accuracy for everyone with age—as people gain experience of judging the ages of others—combined with increased opportunity for reporting errors among male respondents, due to positive heteroscedasticity,^36^ that is, the variance of age disparity rises with age (we see an eightfold increase for men, with no change for women). This heteroscedasticity is due to increasing age disparities for men—mean age disparity for male respondents rose from 2 years at ages 15–24 years to over 5 years by ages over 49 years (in contrast to women, for whom mean age disparity fell). In turn, this pattern of age disparity distributions reflects the fact that young men typically partner with women their own age, but older men partner with younger as well as same-aged women.

These patterns of reporting accuracy and disparity heteroscedasticity have several implications for research and interventions. At a minimum, care should be taken in comparing the accuracy, or level, of age disparities across age and the sexes. For research using age disparities as a predictor, the level of inaccuracy in this analysis—while lower than seen elsewhere—still has the potential to affect results. Misreporting towards zero age disparity is likely to lead to an attenuation of any true relationship. The impact of heaped responses is less clear, although as noted above it is likely to be more significant for dichotomous than for continuous measures of age disparity. A further potential concern is that those engaging in a behaviour of interest may differentially misreport age disparities, if they have a reason to prefer to report larger or smaller age disparities (eg, they feel shame or stigma, or they stand to benefit from reporting a particular level of disparity).

As an example, previous analyses of the association between age disparity and HIV infection in this population—which found no relationship for women aged under 30,^7^ and decreasing risk with increasing age disparity in 30–50 year olds^37^—may have suffered from error in their predictor variable. Understanding the overall impact of age disparity mis-measurement requires a decomposition of the different types of inaccuracy. The very similar results found in these HIV analyses when using age disparity as a continuous or dichotomous variable suggest that heaping did not significantly affect this analysis. Less clear is the impact of many small underestimates of age disparity, although a slight attenuation in the variance of the predictor is unlikely to fully account for the strong null finding in younger women and the strongly negative association seen in older women.

For interventions that target age-disparate relationships, the accuracy and heteroscedasticity patterns of the partner age reports imply that the target audience may need to be set wider than one might expect in order to capture all those relationships truly believed to benefit from an intervention. For example, among women aged under 25 in this sample, sensitivity/specificity for relationships truly 5 years or more age disparate is 74%/90%, using self-reported disparity 5 years or greater. By instead targeting those with a self-reported disparity of 3 years or greater sensitivity for capturing truly five-plus-year disparate relationships rises to 91%, while specificity falls to 51%. Decisions for targeting will, of course, depend on the relative costs and benefits of the intervention for each target group definition.

### Generalisability

In considering the generalisability of our findings, it is instructive to compare this South African study to existing data from Malawi.^23^ The Malawian study found considerably lower sensitivity for women's reports of being in a relationship, where an age disparity greater than 5 years was only 24.3%, compared to 79.6% here, but similar or higher levels of specificity. This pattern suggests that Malawian women rarely report age-disparate relationships correctly, but commonly report non-age-disparate relationships correctly. This difference may well reflect differences in the data at the two sites: the Malawi data included casual as well as marital relationships, and perhaps as a result contained a larger proportion of younger adults. Nevertheless, the large difference in sensitivity combined with similar specificity might reflect lower intentional misreporting in South Africa. Specifically, it seems reasonable to believe that age-disparate relationships may be less stigmatised in the South African setting: age-disparate relationships are common in rural KwaZulu-Natal, and are higher within marriages than in other relationship types,^38^ which may make respondents more willing to offer an accurate assessment of their partner's age when age disparities are large. Additionally, the close links between the surveillance site and the community may have built trust over time, leading to greater honesty.

Alternatively, it may be that reports are more accurate in South Africa because knowledge regarding partner age truly is higher. This is likely given the presence of dates of birth in each person's national identification number, which is displayed in identification books held by almost all citizens. Individuals can then learn a partner's date of birth either through discussion or by seeing their identification book. Partner date of birth is also well-known in this community due to the importance placed on the celebration of partners’ birthdays. As a result, birth dates for almost 90% of individuals in this sample are known to within 1 month. Associations between higher date of birth knowledge and age reporting accuracy have previously been reported in China^15^ and the USA.^16^

These explanations point to several possible limitations to the generalisability of our findings. First, the data set is limited to relatively stable (marital and conjugal) relationships, and may not generalise to other relationship types because reporting accuracy may vary with relationship stability. In particular, it might be expected that more stable relationships have higher accuracy, in which case our findings represent a best-case scenario. Testing whether accuracy varies by relationship type in appropriate data sets would be a useful extension to our work.

Second, since this analysis is of a single setting, it is not clear how geographically generalisable the findings are, given the above discussion of stigma and birth date knowledge. Neither factor seems likely to vary drastically within South Africa; our findings may thus be appropriate for other stable relationships within this country. Elsewhere in sub-Saharan Africa it is likely that misreporting is higher, given that vital registration systems are weaker,^39^ and thus own and partner age reports may both be more accurate than elsewhere (while age heaping in partner age reports is considerable in our data, it is lower than heaping of own age reports in several African and Asian populations^17^ ^40^ ^41^). The differential impact of stigma is less clear: age disparities are common throughout sub-Saharan Africa;^42^ however, the recent increase in anti-sugar daddy campaigns may lead to greater stigmatisation of age-disparate relationships.^7^ As a result, partner age misreporting may increase in the future, reducing the generalisability of these findings over time.

### Strengths and limitations

A major strength of this analysis is the data on which it rests. The collection of conjugal relationships over a 13-year-period and sexual partner reports over 8 years provided almost 14 000 reports of partner age from over 7300 relationships. Missingness in the data set was low for the outcome of interest and for covariates.

There are, however, also several limitations. First, our analysis relies on the accuracy of various data, in particular on self-reported dates of birth, which in turn rely heavily on the national identification number issued to each South African citizen. As highlighted in the Methods section, the relative strength of the South African modern vital registration system means that most young South Africans have had a fixed record of their date of birth since the first month of their life. However, for those non-White citizens (ie, all of this population) over the age of 20, such dates of birth may only have been assigned later, typically around the time of school entry—and may thus be measured with error.

Second, as noted above, generalisability may be limited by the specificities of the study location and the inclusion of only conjugal relationships. Third, despite careful efforts to match conjugal relationships with partners reported through the general health survey, it is likely that some of the matches will be false positives, since we cannot absolutely verify that the actual and perceived age disparities are for the same partner. However, since such measurement error is likely to be random, its effect should be to reduce age report concordance, and thus our results should represent a ‘worst case’ scenario.

Finally, the Bland-Altman and Lin approaches only evaluate validity insofar as the reference data set measure is correct. We may worry that the ‘actual’ age disparity used in this analysis is incorrect, due to respondents either not knowing their own age, or choosing not to provide it. Unintentional error should be limited, given that dates of birth are well known in this area, and the surveillance programme provides multiple opportunities to correct errors. Intentional misreporting should also be limited, since own age is requested as part of demographic surveillance, rather than in the context of sexual behaviour, where social desirability might play a role. In neither case is there a clear reason to expect these misreports of own age to be differential by relationship age disparity, and thus the presence of such errors should again make our findings a ‘worst case’. To the extent that such errors exist, however, our methods evaluate agreement rather than validity.

## Conclusions

Using a very large, population-based, longitudinal cohort, we show that reporting of sexual partners’ ages in rural South Africa is able to identify many truly age-disparate relationships. Accurate reports allow age disparity analyses to rely on sample survey data, rather than needing to interview and link both members of a relationship. This reduces privacy concerns and costs. Furthermore, accurate judgement of partner age allows interventions to focus on providing information regarding appropriate behaviour change, rather than first having to help individuals identify whether they are in age-disparate relationships—in order to ensure that those in the target demographic are aware they are being targeted. Such behaviour change-only interventions are likely to be cheaper and faster.

Age disparities are best measured using partner-based methods where each partner independently reports their age, preferably based on a date of birth taken from vital registration information generated at birth. Given the significant resource cost of such methods, especially in settings where populations are mobile, understanding the accuracy of partner-reported ages is important for research and for interventions. The findings of our analysis highlight the importance of further research into who is, and who is not, able to accurately identify their partner's age, and how this accuracy is connected to subsequent behaviour.
